# Development of a Web-Based Tool for Risk Assessment and Exposure Control Planning of Silica-Producing Tasks in the Construction Sector

**DOI:** 10.3389/fpubh.2020.00371

**Published:** 2020-08-05

**Authors:** Hugh W. Davies, Melanie Gorman-Ng

**Affiliations:** ^1^School of Population and Public Health, University of British Columbia, Vancouver, BC, Canada; ^2^British Columbia Construction Safety Alliance, New Westminster, BC, Canada

**Keywords:** risk assessment, exposure, silica, construction, internet

## Abstract

We describe the development and implementation of a novel, on-line risk assessment tool for respirable crystalline silica (RCS) exposure for use in the construction sector. It was motivated by the introduction of new OHS regulation in British Columbia that allowed for the substitution of exposure measurement data with “objective air monitoring data” collected at “equivalent work operations.” This allowance encouraged the introduction of quantitative risk assessment in a notoriously challenging work environment but it was concluded that without assistance, the typical construction employer would struggle to identify, extract, and interpret validate objective data. The tool described here was based on a continually-updatable RCS exposure database, and a predictive regression model based on the database to generate exposure risk estimates. The model was embedded in an adaptive web-based application that can be run on common platforms. The design followed standard web conventions and features so that no specialized training is required for its use. It was designed to be usable by end-users with varying expertise, including non-OHS experts. Users describe the RCS-dust generating task they will perform, and associated control measures. The tool estimates both uncontrolled and controlled task-based exposure concentrations. Using additional information entered by the user, the on-line tool generates an “exposure control plan” or ECP, a legally regulated document for those undertaking work potentially exposing workers to RCS particulate. The development of the tool was a community-based, tri-partite effort of the local OHS regulator, construction employers, and exposure scientists. In addition to being a practical risk assessment tool, the designers wanted it to function as an educational tool, and that it should explore novel methods for exposure data collection and use. The strengths of this approach include the publicly shared updateable database that encourages continuous improvement and illustrates best practices; and the timely and cost effective collection and sharing of exposure data in a value-added manner. It is however limited to a single task per ECP, and only considers exposure to task operators, and not adjacent workers. Currently in BC, users generate up to 3,900 ECP's per year with the tool.

## Introduction

Occupational exposure to respirable crystalline silica (RCS) is an age-old problem. RCS is a confirmed human carcinogen (1), and exposure is also associated with non-malignant diseases such as silicosis (2) and tuberculosis (3). Interest in silica has been re-kindled in recent years, perhaps due to novel disease clusters in industries such as mining (4), and emerging hazards such as RCS-containing manufactured stone counter tops (5) and fracking (6) as well as by the recent IARC monograph, and revisions that lowered occupational exposure limits for RCS (e.g., 0.05 mg/m^3^ in OSHA and European Union, 0.025 mg/m^3^ for ACGIH TLV®). In a time of generally increasing concern about occupational disease, recent studies of the burden of occupational cancer have more precisely quantified the incidence of occupational disease associated with RCS exposure (7). In Canada, (8) concluded that there are ~600 incident lung cancers annually associated with RCS exposure; few if any of these were previously recognized as occupational nor compensated. In the province of British Columbia, comparison of compensation records with health administration data from hospitals and physicians showed that silicosis was probably far more prevalent than previously thought, with more than 90% of silicosis cases seen by physicians and hospitals not recognized in the BC workers' compensation system (9).

High levels of RCS exposure are particularly prevalent in the construction sector (10) due to the high crystalline silica content of common construction materials, and also the mechanical nature of tasks (e.g., cutting, grinding, and drilling) that are common in construction trades (11). In many parts of the construction sector, there are substantial challenges to measuring and controlling RCS exposure because of the constantly-changing physical work environments. Different contractor and sub-contractor crews come and go at short notice, creating complex management/supervisory relationships. The majority of construction companies can be considered small and medium enterprises (SME's), with minimal OHS expertise and resources. For example, in the British Columbia there are ~200,000 construction workers, but 45,000 registered construction-sector employers (12). Ninety-two percent of companies have <20 employees (13) and each year there are ~5,000 new employer registrations and 5,000 de-registrations with WorkSafeBC, illustrating the fast pace of company turnover (WorkSafeBC, personal communication, 2020).

## Context

In 2013, the British Columbia occupational health and safety (OHS) regulator, WorkSafeBC, proposed amendments to its OHS regulation pertaining to RCS that included a specific requirement for quantitative risk assessment, and the use of exposure monitoring. Demanding exposure assessment was a progressive step, given that there has been a general trend away from quantitative risk assessment for some time, particularly for SME's (14). Reflecting the challenges faced within the construction sector for collecting RCS exposure measurements, the new regulation also allowed for the use of “…objective air monitoring data that was collected during equivalent work operations…” that could be obtained from “…peer-reviewed or scientific studies…” (15). This echoed a similar provision for use of “objective data” provided in the OSHA standard for respirable crystalline silica in the US (16).

While apparently easing the burden on the employer, this “objective data” option posed a new set of challenges that were in many ways more complex than those posed by taking measurements. Focusing on the construction sector (or other SME's) for example, how would the average employer know where to look for suitable peer-reviewed or scientific studies, how to judge “equivalence” of work operations, or how to appraise the validity of the exposure sampling or analysis methods? From the regulator's perspective, how would they evaluate the data extraction effort of the employer, and the validity of employers' interpretations of the data?

In British Columbia, OHS regulations are adopted after a public review and stakeholder consultation. Employers are often concerned about implementation issues such as practicability (i.e., can industry meet these limits) or technical feasibility i.e., is there a validated sampling method–both of which were concerns for the new RCS regulation (17). Supported by the not-for-profit British Columbia Construction Safety Alliance (BCCSA), an initiative was launched in 2014 to find technical ways to assist employers to implement the new regulation, including its quantitative aspects. The idea was to develop a service for employers (and other interested stakeholders) that could assist in identifying appropriate objective exposure data, appraise data validity and provide appropriate data interpretation. The regulator (WorkSafeBC) agreed to participate, and exposure scientists from UBC were engaged to develop scientifically robust strategies and methods for RCS data management, analysis and interpretation.

An initial literature review and environmental scan revealed related similar types of services such as on-line qualitative exposure control plan generators (e.g., Center for Protection of Worker Rights (18), task-specific exposure duration guidelines (19), “first tier” REACH tools (20) and higher-level surveillance efforts such as the European Industrial Minerals Association's “Dust Monitoring Programme” in Europe (21). None of the available tools satisfied the specific requirements of the BC situation, leaving a knowledge gap, and an opportunity for development of an end-user exposure data management tool.

In this community case study, we focus on describing the development, implementation, and maintenance of a novel, multi-part on-line risk assessment tool for respirable crystalline silica (RCS) exposure for use in the construction sector.

## Detail

### Project Design

The project utilized integrated knowledge translation from the outset to make the process transparent and responsive to constituent stakeholders, most importantly the construction industry and the OHS regulators. A tripartite steering committee was formed comprising representatives of (1) the regulator (WorkSafeBC), (2) employers (BC Construction Safety Association, or BCCSA), and (3) exposure assessment scientists (the authors). As needed, this committee drew upon additional expertise in construction safety, construction, and labor practices, web-interface design, data management, and legal and ethical considerations.

The steering committee established the following design objectives for the tool:

1. it was built to assist the construction industry to reduce silica exposures and aid employers in achieving regulatory compliance;
2. it should be usable by end-users with varying expertise, including non-OHS specialists;
3. it should present information in a manner consistent with regulatory-required documents (i.e., “exposure-control plans” or “ECP” as prescribed in BC OHS regulation);
4. it should function as an educational tool, and
5. it should explore novel methods for exposure data collection and use.

It was also agreed that the tool be designed to assist users achieve regulatory compliance but that the simple act of using the tool did not mean that compliance was achieved or guaranteed for the user, i.e., it did not abrogate the obligations of the employer under the BC OHS regulation to understand the hazard, risk, and implement necessary controls.

In addition, the tool was designed to the following general criteria:

1. it should utilize previously obtained high-quality RCS exposure measurement data;
2. the underlying database should be easily and routinely updateable;
3. it should be able to quantitatively predict exposure concentrations for common work scenarios that generate RCS; and
4. it should be able to predict the effect of typically used controls on RCS exposure levels.

### Exposure Data

A literature review of peer-reviewed and gray literature (e.g., non-peer reviewed technical reports) was undertaken to identify sources of published respirable crystalline silica exposure data. We identified a recent systematic review on the same topic (22) and therefore we restricted our literature review to post-2004 publications. We also sought out research and industry RCS exposure data holdings, government/regulator RCS data holdings and finally, we updated with the authors' own exposure data collected as part of RCS projects that were underway in the Provinces of British Columbia and Alberta. Validity of retrieved exposure data was assessed using the following criteria: (i) analyte must be respirable crystalline silica; (ii) a standard method must be used (e.g., NIOSH 7500 [including 8 LPM PPI version], NIOSH 7602, OSHA ID-142); (iii) it provides adequate description of key sampling parameters (e.g., flow rate, duration); (iv) it confirmed adequate original QA/QC (e.g., calibration, field blanks, sample volume, etc.); (v) it contained sufficient supplementary data to allow assurance of “equivalence” of operations as per WorkSafeBC OHS regulation; and (vi) it was personal sampling. Some of the data retrieved were in the form of summary statistics (e.g., means and standard deviations). To make summarized data available to the tool, we de-aggregated summary statistics by generating simulated discrete observations using the technique of Lavoué et al. (23); also Sauvé et al. (24) who previously used this technique with Quebec RCS data in our database).

The initial literature review and environmental scan found 16,115 silica-related exposure measurements (Table 1). Of these, 73% were from the Quebec literature review (22), 23% from the updated literature review by the authors, and 2% each from new data measurements and from measurement data collected from local industry. Of the 16,115 measurements, 27% (~4,500) met the validation criteria, and were made available for modeling. Approximately 15% of values were non-detects. Values below the limit of detection were replaced with ½ LOD.

**Table 1:** Number of sources of RCS exposure data and exposure data points in the final database used for analysis.

**Source of data**	**Sources/publications**	**Exposure measurement data points**	**Exposure measurement data points meeting quality control criteria**
Beaudry et al. (22) database	115	11,845	3,487
Updated literature review (literature published 2008–2014)	35	3,625	680
Shared by Canadian companies	7	58	18
Shared by Canadian government agencies	1	264	176
Exposure monitoring campaign by the authors at BC worksites	N/A	343	119^*^
Total	158	16,135	4,480

* *All samples were analyzed for respirable dust, respirable quartz, and respirable cristobalite. Respirable dust was excluded from final analysis, and quartz, and cristobalite measurements were consolidated as respirable crystalline silica, by adding masses of polymorphs and dividing by total air volume sampled*.

**Figure 1:**
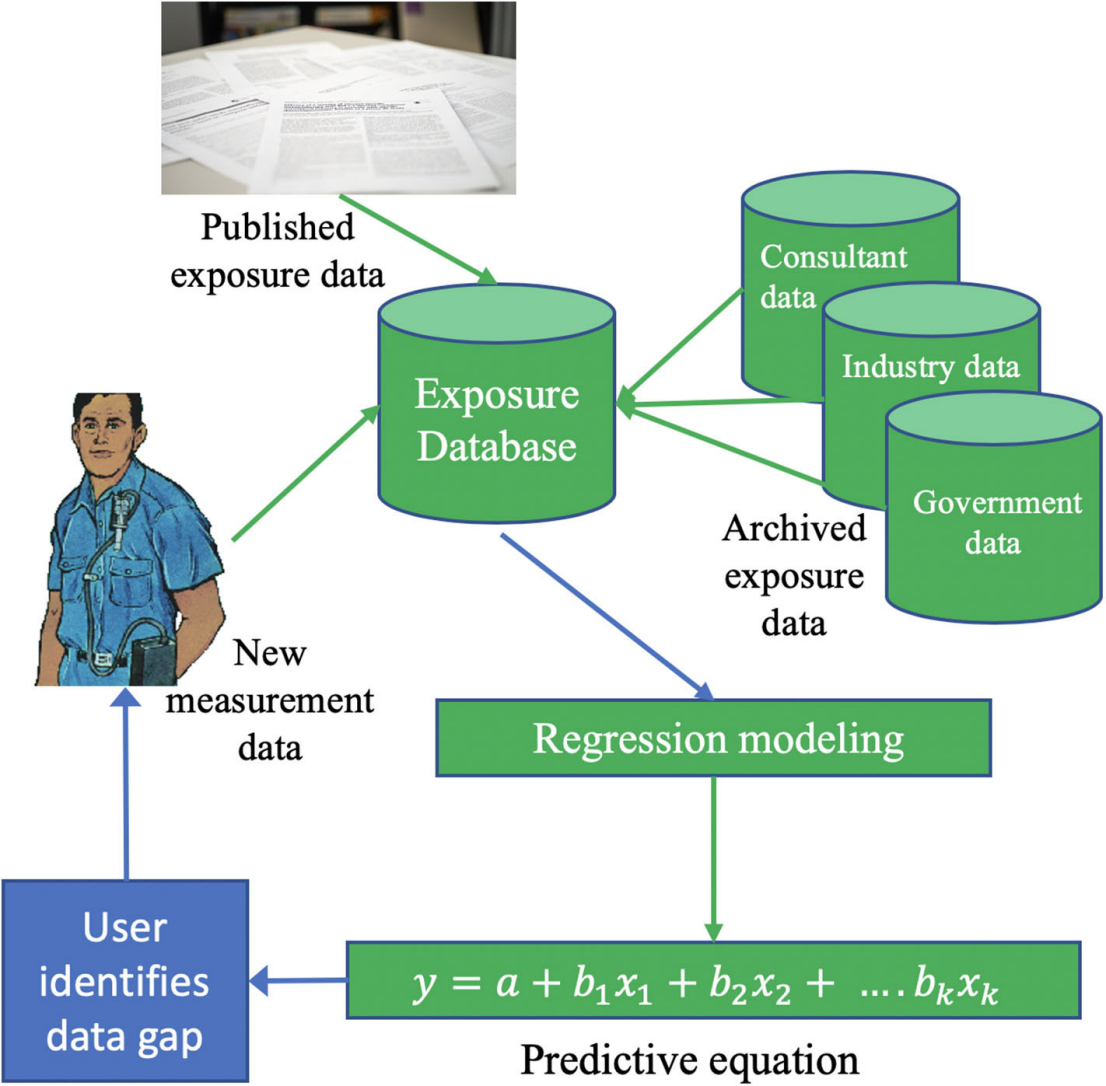
Exposure data capture and flow in final design. After the initial population of the exposure data base from archived data, it can be easily and routinely updated with newly acquired exposure data. The need for exposure monitoring is indicated where work is being done and no data exists in the database to permit modeling and exposure estimation. Initially, new exposure data collection was subsidized to encourage timely acquisition.

In the final design (Figure 1), the database is to be continually updated by routine capture of data though literature review updates, ongoing “harvesting” of archived data from industry and government sources, but also new data measurement. The database and new data capture are managed by BCCSA. New measurements can be targeted to data-poor areas by on-line tool users who report data deficiencies in the tool. Because they are seeking data for a specific silica process, they presumably undertake the task and are therefore in a position to measure and submit data to the RCS database. To enable this, local consulting companies were recruited and provided standardized data collection forms to ensure QA/QC criteria are met.

### Modeling

To focus our efforts, we developed a “Common Silica Process” or “CSP” construct upon which to base exposure estimates. CSPs combine task (e.g., grinding, drilling) with material (e.g., concrete, asphalt,) and tool (e.g., jackhammer, hand-held power saw) to describe an RCS generating work process. To identify high priority CSPs in BC we arranged a focus group of six local construction industry employer representatives. They identified 24 CSP's that were focused on initially (Table 2). Additional CSPs were added following development of the tool during the pilot testing phase, and the design of the tool allows for new CSPs to be added when identified by users.

**Table 2:** British Columbia common silica processes (CSPs) included in the respirable crystalline silica exposure model by material.

**Material**	**Common silica process (CSP)**
Asphalt	Cutting asphalt with walk- behind saw
	Milling asphalt with milling machine
Concrete masonry unit	Cutting concrete masonry units with table saw
	Cutting concrete masonry units with powered portable saw
Concrete	Cutting concrete with saw
	Coring concrete with coring machine
	Drilling concrete with electric hammer drill
	Grinding concrete with angle, surface, right angle, or flat grinder
	Grinding concrete with counterbalanced ceiling grinder
	Scarifying or bush hammering concrete
	Loading concrete mixer truck
	Breaking concrete with jackhammer
Shot-crete	Spraying shot-crete with compressed air mixture
Ceramic tiles	Cutting ceramic tiles with portable powered tile saw
Rock/sand/earth	Mechanized moving of rock/sand/earth with heavy equipment
	Manual moving of rock/sand/earth
	Crushing and processing rock/sand/earth with a stationary or mobile crusher
Marble/granite	Cutting marble and/or granite with a powered saw
Cementicious material	Mixing and pouring cementicious material
Drywall	Cutting drywall with a saw
	Grinding drywall with a sander or grinder
Mortar	Tuck point grinding
Fiber cement board	Cutting fiber cement board with a portable saw
Various	Demolition of rock or concrete structures
	Manual sweeping of rock or concrete construction dust

Multiple regression analysis was used to develop predictive models to be used in the tool. We selected variables for analysis based on the variables that were found to be significantly related to RCS exposure by Sauvé et al. (24) task-based analysis of the Beaudry et al. (22) database. Variables also had to have relevance to the British Columbia construction industry, and whose values would be expected to be readily known by the end-user when using the predictive model to produce exposure estimates.

The final model selected for exposure estimation was a parsimonious multiple linear regression model with exposure level (in mg/m^3^) as the dependent variable, and the following predictor variables: sampling duration (minutes); common silica process (combines task, tool, and material variables); industry sector (e.g., residential, industrial, civil); project type (e.g., new, renovation, or demolition); work environment (indoor or outdoor, restricted space, confined space); sample duration, and geographic region. In addition, the following engineering controls were incorporated: local exhaust ventilation, local exhaust ventilation integrated with tool, material wetting, water-spray integrated with tool, and separation from source (e.g., enclosed cab). In the case of separation from source there was insufficient data to model so exposure reductions were estimated as a factor (25%) derived from published literature (25). Final estimates were discounted by 50% if user states that exposure duration was <4 h (based on similar allowance in the OSHA ruling).

To reflect underlying data variability while still only presenting a single point estimate for simplicity, the user is given the upper 95th confidence interval, estimated using Monte Carlo simulation based on 1,000 values within a distribution defined by the variable coefficient (as the mean) and the variable standard error (as the standard deviation) for each model variable.

### Web-Based Interface

**Figure 2:**
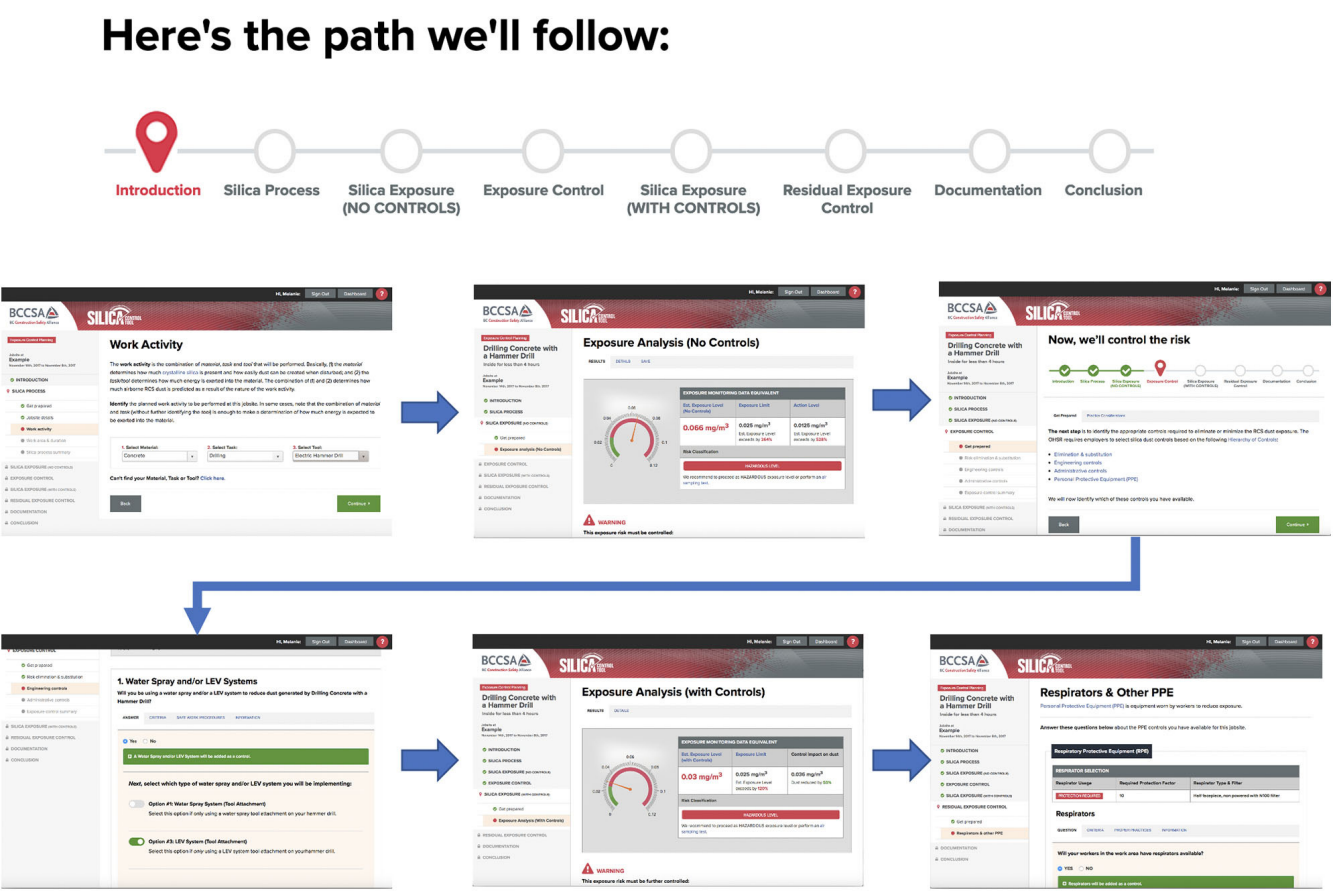
Framework of web-application showing (top) the process flow schematic presented to the user as they progress through generating an ECP; selected screen shots to illustrate design and key steps in session.

The user interface was designed as an adaptive web-based application that could run on all major platform types (e.g., computer, tablet, smartphone). The “app” followed standard web-design conventions and features so that no specialized training was required for its use. The app was given multiple levels of educational material including short definitions, FAQ's, longer explanatory pages, and links to PDF's and external web pages for detailed information. Educational information included topics such as tool usage, occupational hygiene basics, health hazards of RCS, the control hierarchy, control descriptions, etc. Educational materials are accessed by hypertext links, and through tabs on higher-level pages. The principal components of the on-line tool are outlined in Figure 2, including the “process flow.”

A user “session” is designed around the production of an exposure control plan (ECP) for a specific work task. To generate an ECP, users sign in, enter or modify company and job-specific identifiers and descriptors, then describe the work for which the risk assessment and ECP is to be generated. Users have accounts (and accounts can have multiple sub-accounts) and the tool stores user-specific identifier data (contact names, addresses, company logos, etc.) as well as archived ECP's previously produced by the tool for that user. This provides accountability and auditability, as well as speeding up new ECP development, by allowing copying and editing of archived ECP's.

For the quantitative exposure estimates, users enter values for required predictor variables as well as details of the controls they propose to use. The tool provides examples for variables, such as work environment, to help user select valid values. The web-based interface uses the latest version of the predictor model to make exposure estimates for both uncontrolled and controlled scenarios. A new predictor model is generated and uploaded to the tool whenever there is significant new exposure data added to the underlying exposure database.

**Figure 3:**
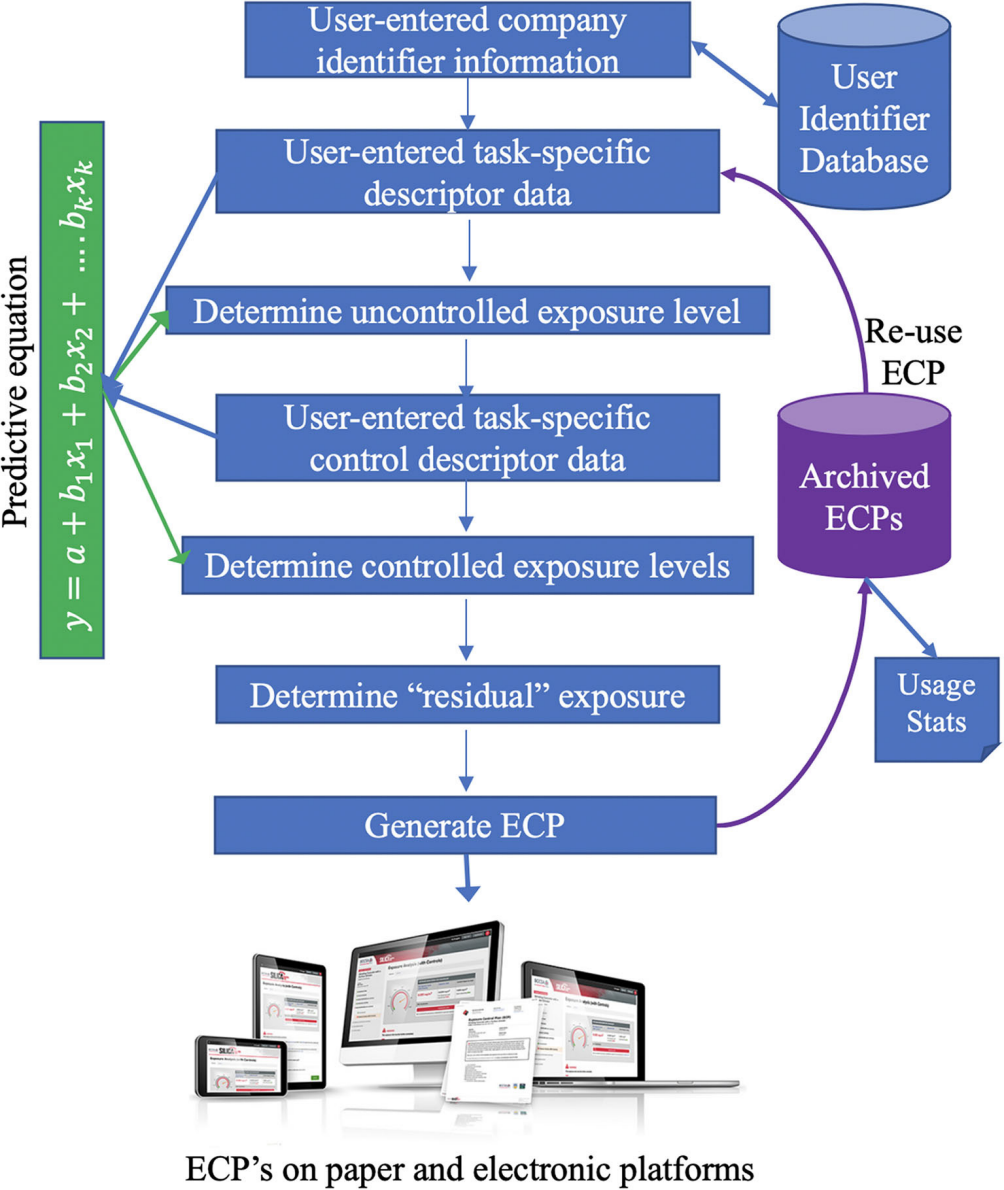
Data flow and gross logic of on-line risk assessment tool. Company identifier data is used for accountability and in report generation. Archived ECP's can be re-used as the basis for new ECP's to reduce repetition. Users enter descriptive information about the task to be performed and the predictive model estimates exposure concentrations for the uncontrolled and controlled exposure scenario. Users can iterate this step to do “what-if” estimates. Final product is an “exposure control plan” that meets regulator's requirements; in electronic form it can be easily distributed to work crews.

The principal internal operations of the tool are shown in Figure 3. The online interface comprises 6 distinct functions:

1. Accounting: The tool is available to all BC employers. New user accounts are set up by providing basic company identifying information. This data is presented on the title page of each exposure control plan (ECP) generated. Each user's data is kept confidentially and not accessible to other users. Additional job-level data can be specified for individual ECP's (job address, supervisor name, etc.);
2. ECP archive: Once an ECP is generated it is stored on-line and can be recalled, copied, edited, printed, or deleted. Each ECP is “version-stamped” with the database version used to generate exposure estimates;
3. Task data capture: several web pages are used to capture descriptive data to be used in predictive modeling (material, task, tool, environment, duration, etc.)
4. Calculations: The predictive model is embedded in on-line code; exposure estimates are calculated based on the users' inputs. The estimate shown is the upper 95 %'ile. Two estimates are provided, one without controls and one with selected controls in place. The intermediate results are shown in a visual “speedometer” form (Figure 2) and the user can cycle iteratively, to perform “what-if” trials to examine the effect of varying exposure control measures. Pre- and post-control predicted exposure levels are compared to an OEL; if there is a “residual” over-exposure following specification of controls, the tool will recommend appropriate respiratory protection to achieve compliance.
5. Education: the on-line interface is embedded with hypertext links throughout to technical definitions, FAQ pages, educational pages, checklists, etc. to support the user. External links to regulations, and to research is also coded.
6. Outputs: the tool produces a document that is in compliance with WorkSafeBC requirements for risk assessment and exposure control plans. The document is in PDF format and can be printed, or stored and shared electronically with employees and the regulator on request.

The primary output of the tool is the multi-page ECP, designed to meet the procedural and content requirements of the BC regulator. The ECP includes: risk assessment details, control information (engineering, administrative, and PPE) and relevant supporting text is bundled into a PDF that can be printed, or viewed on most web-enabled device for ease of distribution and use.

### Public Release

The tool went through multiple pilot-test phases with small groups, with feedback being incorporated into the tool. The tool was then released to the public in May, 2017 as the “Silica Control Tool.” All companies registered with WorkSafeBC have automatic access. Others may request trial licenses by contacting silicatool@bccsa.ca. Roll-out involved public advertising (e.g., https://youtu.be/pOHf8WSPbAs), presentations at local health and safety and industry conferences, as well as knowledge translation exercises with local industry groups. Because the tool used common web-based app conventions no special training was required. In-depth orientations were provided to BC regulatory occupational health officers who play a key role in prevention activities so that officers would be familiar with the strengths and limitations of the on-line tool and would be better able to interpret its use during on-site inspections. British Columbia has a large contingent of Occupational Hygiene Officers. In 2018 they spent 230,000 h in inspection activities, and we would expect this level of effort and knowledge should aid the effectiveness of this effort (12). The BCCSA provided on-call technical support and in the initial phase they also financially supported discounted exposure measurement campaigns to help fill gaps in the exposure database. In the first 3 years (2017–2019), the number of on-line sessions climbed from 6,300 to 9,400, respectively, while the number of ECP's started rose from 1,700 to 3,900 per year.

## Discussion

There is widespread agreement among occupational hygienists of the importance of quantitative exposure measurement (26). Occupational hygiene practitioners use exposure data for compliance monitoring, but also control selection, intervention evaluation, surveillance of trends in exposure, and for education purposes. Yet, since the 1990's, exposure data collection by regulators in Canada (and elsewhere) has sharply declined (27). Increased emphasis has been placed on qualitative exposure assessment such as occupational exposure banding (14). Where data does exist, data interpretation techniques remain complex, and largely in the domain of a subset of industrial hygienists (28). Particularly, there are few if any quantitative exposure assessment resources for the non-OHS expert. The on-line tool described in this paper presents a novel approach to exploit archived exposure data to assist the non-expert in exposure risk assessment and control planning. The tool has potential benefits in providing timely risk assessments for complex work environments, ease-of-use for non-experts, cost-effective data collection, data mobilization, and to drive continuous improvement in terms of controls. It is one example of a way that independent companies can contribute small volumes of anonymized data and receive a value-added benefit from the large, aggregate, centralized database.

### Practical Considerations and Significance

A key aspect of the tool's design is the continual update of the database. The tool does not generate an exposure estimate if objective data is not available. Users seeking risk assessment for tasks for which objective exposure data does not exist are directed to take exposure measurements using standard methods and are requested to contribute results to the shared database. To enhance participation in the current project, the employer-funded safety association BCCSA offset costs associated with RCS sampling. OHS regulators in other Canadian Provinces (Alberta, Manitoba) have also funded projects to collect RCS data to strengthen the database regionally and for validation purposes.

Periodic update of the database means that it should always reflect best practice and thus serve to encourage a “continual improvement” approach to reducing occupation exposure, and disease, incidence (29). As an example, during the development of the tool, we identified local contractors who were using a hollow concrete drill bit and called “virtually dust-free” by the manufacturer (Hilti, Mississauga, ON). Designed to be attached to a vacuum, dust is removed from the tip of the drill as produced, removing the requirement to clean the hole after drilling and making a better “product.” However, a secondary benefit is reduced levels of dust exposure in the air (30). By taking RCS measurements during hollow-point drill use and adding them to the RCS exposure database, all other construction employers using the on-line tool are made aware of this new technology in a timely manner. This important information is also available to other stakeholders such as regulatory officers, who can make decisions on appropriateness of controls in light of newly-available technology. This is particularly important given that RCS is a confirmed human carcinogen and thus an ALARA (“As Low As Reasonably Achievable”) substance. One definition of “reasonably achievable” is that it is being demonstrated by others; thus in this example the use of hollow-point drills should be more quickly and more effectively identified as “best practice” and hopefully more widely adopted.

Compared to a more “stable” work environment (e.g., manufacturing sector) construction presents an ever-changing work environment and traditional exposure assess strategies based on random samples drawn from homogeneous exposure groups (31) are not so straightforward. Employers are likely reluctant to order expensive exposure measurements when turn-around time for RCS analysis is several days and a construction worksite will have significantly changed before a risk assessment is completed. The sheer number of SME's in the construction sector would mean the generation of large volumes of small-numbers of samples under a traditional approach. Using the model/tool approach and a shared database means that sampling will be more cost effective, and available in a timelier manner. Further, it avoids duplication of effort, and the creation of small isolated silos of exposure data. In BC exposure measurements are no longer collected by the regulator, even if the regulator ordered them to be taken. Data “lost” in this fashion has been shown to very expensive to recover (32) but this type of tool will hopefully increase the rate of capture of exposure measurements. In general, we have observed industry more willing to share data when (a) the data custodian is an independent entity and not the regulator and (b) there is a clear value-added product.

Development of tools such as the one described in this paper that capture, share and utilize data on a shorter time scale perhaps have the potential to change the dynamic around occupational exposure databases. In the past these large (often national) databases have been very “static,” and used primarily for research and in many cases such as Canada contain largely historical data (27). Capturing contemporary data and providing tools to “value-add” in widely available and accessible ways may re-vitalize exposure data collection that has been declining for over 2 decades. Adding more contemporary exposure data to an RCS exposure data repository will also contribute data for ongoing exposure surveillance (trend analysis) as well as providing data for epidemiological and related (burden of disease, etc.) studies.

Overall there has been good acceptance of the tool, and it has been a strong point of focus in the regulator's efforts to strengthen prevention of RCS exposure. BC employers are directed to the tool in the BC OHS Guidelines, that are designed “to help with the application and interpretation of sections of the *Occupational Health and Safety Regulation”* (33). It has certainly meant that employers and employees have a better understanding of exposure levels and are able to see objective evidence of exposure and as well, control effectiveness. Currently the tool is only in use in BC but other Canadian and US jurisdictions have expressed interest; the tool itself is easily adapted to different jurisdictions as geographic region is accounted for in the underlying model, and other jurisdiction specific parameters (like OEL) can be readily modified.

### Conceptual or Methodological Constraints

The implementation of this on-line RCS risk-assessment tool was a response to a specific challenge posed by the construction industry's need for tools to make a new regulatory intervention feasible. It was time and resource-constrained, and not surprisingly has a number of limitations. Despite the apparent large amount of RCS exposure data found in the literature, remarkably little was suitable for use in regression modeling and an even smaller number relevant to the priority common silica processes (CSPs) we identified in our focus groups. The resulting level of discrimination between similar tasks (such as saw diameter) or within controls (such as water flow rate) was less than originally hoped, although for certain tasks (like grinding) several tool types could still be distinguished. In the future, increasing amounts of data should increase the level of detail possible. Since the original implementation of the tool there has been an ongoing effort to add data, and the general response from employers, industry and other stakeholders has been positive. Additional data will also improve validity of the exposure estimates, expand the number of tasks covered, and reduce variability. We validated the model against measured RCS exposure data (*N* = 65) for 7 CSP's. We compared modeled geometric mean and 95th percentiles to measured data. The modeled exposure estimates correlated moderately well with measured exposure measurements, with 95th percentile modeled exposure estimates overestimating exposure levels on average (average of ratio of modeled to measured = 1.90). In the validation, 95th percentile model estimates were conservative 55% of the time. When compared to measured data our silica model compares favorably to the European models with a correlation coefficient of 0.50. Further details of validation will be published separately.

The tool only provides exposure estimates and ECP's for one silica control process (task/material/tool) at a time. If needed, more complex scenarios must be broken down by the user and treated as discrete tasks. We did not consider this a major limitation as typical engineering controls are task specific, and often sub-contractors do a limited set of tasks. Also, the tool estimates exposure levels on a task basis, i.e., the model doesn't consider duration of exposure. Therefore, to estimate and 8-h average exposure requires a task-based time-weighted averaging approach. Note that the steering committee did agree to add a 50% reduction for work durations of <4 h based on the OSHA standard, but this is admittedly a crude correction; it requires validation and may need to be revised.

The tool only estimates exposures for the worker conducting the task, as most of the original data from which predictive models were built were personal exposure measurements of the worker performing the task. The ECP does include general language about recognizing the need to minimize RCS exposure for others working in the vicinity of the task. The tool does not take into account other RCS-generating tasks that may be going on around the worker at the time the sample was taken since we have no information about that. The tool asks users to “check” administrative controls that are in place but does not use them in quantitative exposure estimations; it does print them in the resulting ECP.

## Lessons Learned for Future Consideration

The Silica Control Tool offers a novel tool design aimed at “mobilizing” archived exposure data to support risk reduction in the construction industry. It met the primary design objectives, most importantly retaining a quantitative, measurement-based approach. It has provided a platform for education around the RCS problem and mitigating controls; it is transparent and accountable, and it has generated a new body of exposure data that can be used for wide-scale exposure surveillance. Importantly it has received wide support from different stakeholders including employers and regulators and become a focal point for silica risk reduction efforts in British Columbia. The tool uses modeling, and as the aphorism goes, models are always wrong, but some may be useful. We've addressed this concern by providing the upper 95th percentile of the estimate which is inherently conservative; we have validated the model estimates and found the model behaves comparably with other modeling efforts; and we are striving to add data to the underlying database to improve exposure estimates. We believe the alternative (end-users locating, analyzing and interpreting objective data on case-by-case basis) would lead to less accurate assessments. Our interaction with users also suggests the effect of having quantitative estimates (of their own specific exposure scenarios) has greatly helped them understand the risk, by seeing how differing conditions influence RCS exposure levels–and in some cases how *little* difference controls sometimes make; for example measurement data shows dust extraction systems *lower* but do not completely *remove* the risk). End-users can also examine the quantitative difference between different control strategies, and see what controls represent “best practice.” This demonstrates the power of measurement data beyond that of simply assessing compliance. For the future, the authors are undertaking improvements to the modeling/prediction “engine” by incorporating Bayesian statistics to both (1) provide different kinds of risk information to the end user, and (2) provide approaches whereby small amounts of contemporary data can be more effectively combined with larger volumes of older data to produce better exposure estimates. The tool is seen as most beneficial to industries such as construction where it is hard to obtain exposure measurements, but there has been some interest in expanding to other common exposures in the construction sector, such as noise and other chemicals.

Overall, occupational hygiene could be said to lag the widespread innovation in data-usage trends, and there is a dearth of tools designed to enable and enhance exposure data collection, data sharing, data interpretation. Advances in measurement techniques mean that exposure data is ever easier to obtain, and in general, both data management and analysis are increasingly cheap and increasingly sophisticated. Simultaneously, evolving open-data policies encourage data sharing to enhance accountability transparency and to meet the needs of stakeholders seeking evidence-based solutions. We hope that the tool described in this paper provokes others to develop similar or more advanced tools to better mobilize data.

## Data Availability Statement

The raw data supporting the conclusions of this article will be made available by the authors, without undue reservation.

## Author Contributions

HD and MG-N were members of the RCS On-line Risk Assessment Tool project steering committee and involved in all aspects of the project from initial design through build and implementation. HD led the writing of this manuscript. All authors contributed to the article, its revision, and read and approved the submitted version.

### Conflict of Interest

The authors declare that the research was conducted in the absence of any commercial or financial relationships that could be construed as a potential conflict of interest.
